# 2-Amino-5-chloro­pyridin-1-ium barbiturate dihydrate

**DOI:** 10.1107/S2414314626003512

**Published:** 2026-04-10

**Authors:** Thankappan Ramalakshmi Anantheeswary, Sundaramoorthy Gomathi, Jeyaraman Selvaraj Nirmalram, Franc Perdih

**Affiliations:** a Department of Chemistry Periyar Maniammai Institute of Science & Technology, Thanjavur Tamilnadu-613403 India; b Department of Chemistry, Government Arts and Science College, Karambakudi, Pudukottai, Tamilnadu-622302, India; c Faculty of Chemistry and Chemical Technology, University of Ljubljana, Slovenia; University of Aberdeen, United Kingdom

**Keywords:** crystal structure, mol­ecular salt, 2-amino-5-chloro­pyridinium cation, barbiturate anion

## Abstract

In the title hydrated salt, C_5_H_6_ClN_2_^+^·C_4_H_3_N_2_O_3_^−^·2H_2_O, the asymmetric unit contains two cations, two anions and four water mol­ecules of crystallization. In the extended structure, the barbiturate (2,4,6-trioxo-1,3-diazinan-5-ide) anions form a wave-like supra­molecular [001] chain decorated by the cations, mediated by numerous N—H⋯O hydrogen bonds. Adjacent chains are cross-linked by [010] chains of water mol­ecules featuring four- and six-membered rings and five-membered carboxyl­ate aggregates *via* O—H⋯O hydrogen bonds.

## Abstract 2

The extended structure of the title hydrated salt is consolidated by numerous N—H⋯O and O—H⋯O hydrogen bonds.

## Graphical abstract

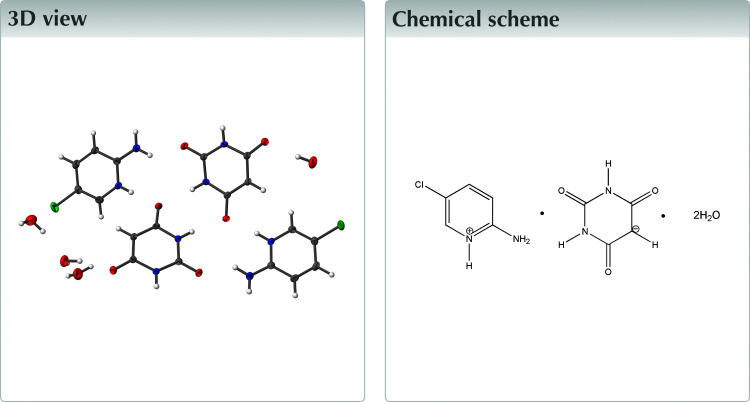

## Structure description

The crystal structure of 2-amino-5-chloro­pyridine, C_5_H_5_ClN_2_ (Kvick & Backéus, 1974 ▸) and its diverse salts with di­carb­oxy­lic acids (Jayanalina *et al.*, 2015 ▸), aromatic acids (Hanif *et al.*, 2020 ▸) and other inorganic anions such as nitrate (Zaouali Zgolli *et al.*, 2009 ▸), phosphate (Akriche & Rzaigui, 2005 ▸), sulfonate (Jagan & Boopathi, 2020 ▸) and tri­fluoro­acetate (Hemamalini & Fun, 2010 ▸) have been extensively studied, highlighting its diverse hydrogen-bonding inter­actions. Barbiturates derived from barbituric acid (C_4_H_4_N_2_O_3_) play a significant role in biological systems (Hueso Ureña *et al.*, 2003 ▸). Research on barbiturate salts and co-crystals demonstrate that hydrogen bonding is a key driving force for structure and property modulation.

Hydrogen bonding not only increases the reactivity and electrophilicity of barbiturates (Bauer & Spange, 2010 ▸), but also governs their supra­molecular organization in host–guest systems and polymeric assemblies, particularly those involving Hamilton-type receptors (Chang & Hamilton, 1988 ▸). Thus hydrogen bonding acts as the primary driving force behind barbiturate self-assembly and functional behaviour and indicates its significance in supra­molecular chemistry, materials science and biomimetic design. As part of our studies in this area, we now report the synthesis and structure of the title hydrated salt, C_5_H_6_ClN_2_^+^·C_4_H_3_N_2_O_3_^−^·2H_2_O (**I**).

**Figure 1:**
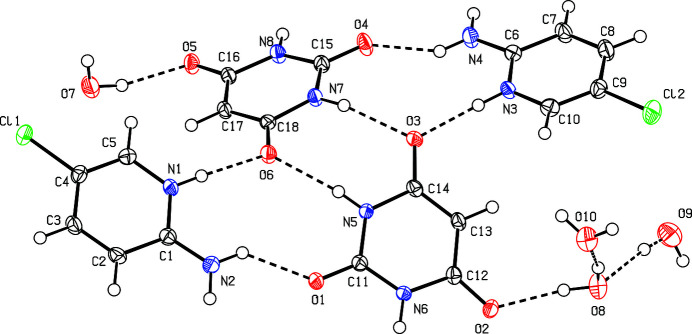
The mol­ecular structure of (**I**) with displacement ellipsoids drawn at the 50% probability level.

The asymmetric unit of (**I**) consists of two crystallographically independent 2-amino-5-chloro­pyridinium cations, two barbiturate anions and four water mol­ecules of crystallization in space group *P*2_1_/*c*. The proton acceptance from the barbituric acid occurs at the pyridine ring N atoms of the cations (Fig. 1 ▸) which is evident from the widening of the C1—N1—C5 and C6—N3—C10 bond angles [122.67 (13) and 122.84 (14)°, respectively] compared to the unprotonated mol­ecule, in which the bond angle is around 118° (Anantheeswary *et al.*, 2024 ▸). The deprotonation of the barbituric acid occurs from the active methyl­ene groups (atoms C13 and C17) driven by the electron withdrawing carbonyl group at both sides and this is supported by the *sp*^2^ hybridization at these atoms in (**I**) implied by the C—C—C bond angles [C12—C13—C14 = 121.55 (14); C16—C17—C18 = 121.61 (14)°]. Overall, both barbiturate anions display a nearly planar six-membered ring: the deviations of the atoms from the mean plane are small (±0.025 Å and ±0.013 Å for the O1 and O4 anions, respectively). These structural parameters align well with known data for barbiturate systems (Gelbrich *et al.*, 2015 ▸) and indicate that the barbiturate anion in (**I**) adopts a stabilized, delocalized and nearly planar conformation.

**Figure 2:**
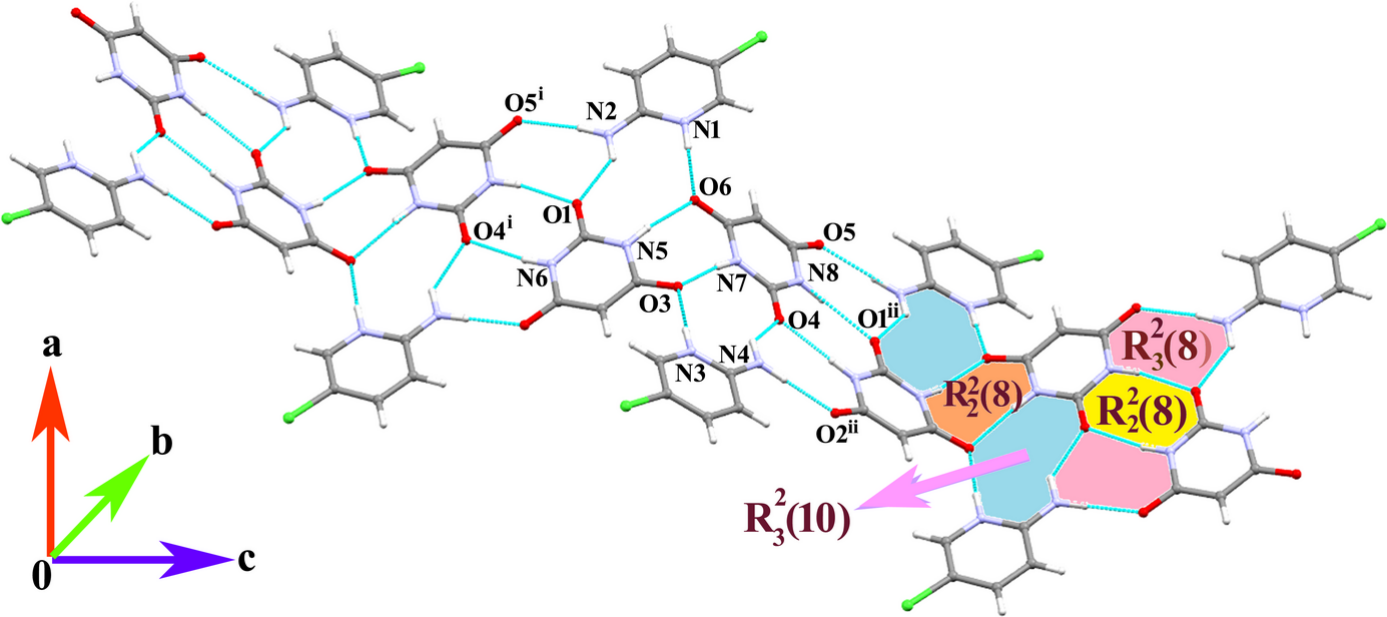
A view of a wave-like supra­molecular chain built up from N—H⋯O hydrogen bonds [symmetry codes: (i) *x*, 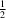 − *y*, −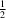 + *z*; (ii) *x*, 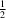 − *y*, 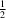 + *z*].

**Figure 3:**
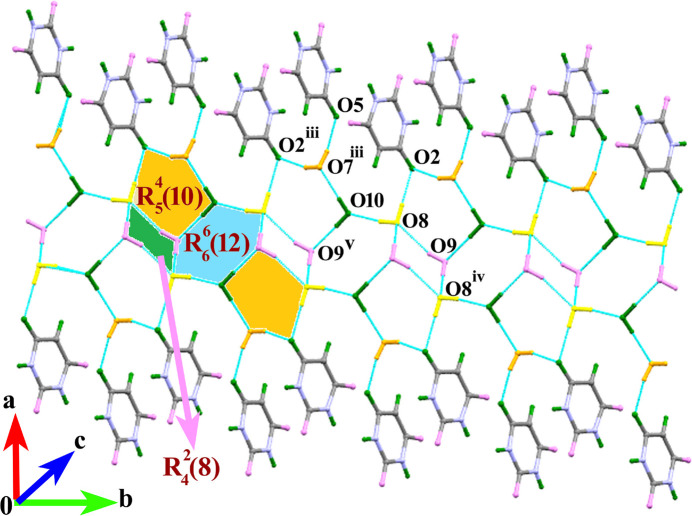
A water chain extending along the *c-*axis direction cross-linking the supra­molecular chains [symmetry code: (iii) 1 − *x*, 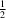 + *y*, 3/2 - −*z*].

In the extended structure of (**I**), the anions are linked by pairwise N—H⋯O hydrogen bonds into wave-like [001] ribbons, with the O1 and O4 anions alternating in the chains. Atoms N5 and N7 (donors) and carbonyl oxygen atoms O3 and O6 (acceptors) form one pairwise linkage and atoms N6 and N8 (donors) and O1 and O4 (acceptors) the other, which leads to two distinct 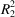
(8) ring motifs (Table 1 ▸). The [001] chains are decorated by the cations: the C1 cation links to atoms O1, O5 and O6 in the anions and the C6 cation to O2, O3 and O4 *via* strong N—H⋯O hydrogen bonds to render 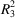
(10) ring motifs. Taken together, these hydrogen bonds lead to propagate a wave-like supra­molecular ribbon as shown in Fig. 2 ▸. Adjacent anion/cation supra­molecular sheets are connected by four- and six-membered [010] tape-like arrays of water mol­ecules which form various O_w_—H_w_⋯O_w_ and O_w_—H_w_⋯O_c_ (w = water, c = carbon­yl) hydrogen bonds. This arrangement has a close resemblance to the water tape *T*4(2) and *T*6(2) motifs in the systematic classification of hydrated organic crystal structures reported in the literature (Infantes & Motherwell, 2002 ▸). In addition, the inter­action between the water mol­ecules O7, O9 and O10 and the carbonyl O2 atom of the anion leads to an 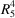
(10) ring motif. The existence of the four- and six-membered water loops and five-membered carbon­yl–water loops generates two different *DDAA* (*D* = donor, *A* = acceptor) hydrogen-bonded arrays with ring motifs of 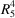
(10), 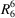
(12) and 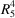
(10) and 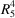
(10), 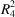
(8) and 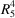
(10), along the *a* axis direction as shown in Fig. 3 ▸.

A possible offset aromatic π–π stacking inter­action between barbiturate rings [*Cg*1⋯*Cg*2^vi^; symmetry code: (vi) 1 − *x*, −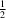 + *y*, 3/2 − z; *Cg*1 = C11–C14/N5/N6 centroid; *Cg*2 = C15–C18/N7/N8 centroid] occurs with centroid-to-centroid and perpendicular distances of 3.7978 (9) and 3.4530 (6) Å, respectively. However, the large slippage angle of 30.5° suggests that this inter­action is weak.

## Synthesis and crystallization

The title compound was synthesized by mixing 20 ml ethanol:water (1:1 *v/v*) solutions of 2-amino-5-chloro­pyridine (0.25 mmol) and barbituric acid (0.25 mmol) and the resulting clear solution was then warmed over a water bath for 20 min at 353 K. The solution was then allowed to cool to room temperature and after a few days, colourless crystals of (**I**) were separated out from the mother liquor.

## Refinement

Crystal data, data collection and structure refinement details are summarized in Table 2 ▸.

## Supplementary Material

Crystal structure: contains datablock(s) global, I. DOI: 10.1107/S2414314626003512/hb4559sup1.cif

Structure factors: contains datablock(s) I. DOI: 10.1107/S2414314626003512/hb4559Isup2.hkl

Supporting information file. DOI: 10.1107/S2414314626003512/hb4559Isup3.cml

CCDC reference: 2543974

Additional supporting information:  crystallographic information; 3D view; checkCIF report

## Figures and Tables

**Table 1:** Hydrogen-bond geometry (Å, °)

*D*—H⋯*A*	*D*—H	H⋯*A*	*D*⋯*A*	*D*—H⋯*A*
N1—H1⋯O6	0.87	1.85	2.7207 (18)	176
N2—H2*A*⋯O1	0.89	2.02	2.7461 (18)	138
N2—H2*B*⋯O5^i^	0.89	1.87	2.7610 (17)	180
N3—H3*A*⋯O3	0.86	1.83	2.6863 (18)	177
N4—H4*A*⋯O4	0.87	2.00	2.7248 (18)	139
N4—H4*B*⋯O2^ii^	0.89	1.97	2.8606 (17)	173
N5—H5*A*⋯O6	0.87	2.02	2.8786 (17)	173
N6—H6⋯O4^i^	0.89	2.03	2.9005 (17)	170
N7—H7*A*⋯O3	0.87	2.06	2.8985 (17)	163
N8—H8*A*⋯O1^ii^	0.88	2.01	2.8762 (16)	172
O7—H7*B*⋯O5	0.86	1.92	2.7581 (16)	164
O7—H7*C*⋯O2^iii^	0.82	2.13	2.9155 (18)	160
O8—H8*B*⋯O10	0.86	1.89	2.739 (2)	167
O8—H8*C*⋯O2	0.89	1.91	2.7810 (16)	165
O9—H9*A*⋯O8	0.86	1.93	2.7864 (18)	168
O10—H10*B*⋯O7^iii^	0.87	1.96	2.8287 (18)	175
C10—H10⋯O7^iii^	0.95	2.44	3.321 (2)	153

Symmetry codes: (i) 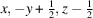; (ii) 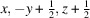; (iii) 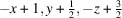.

**Table 2:** Experimental details

Crystal data	
Chemical formula	C_5_H_6_ClN_2_^+^·C_4_H_3_N_2_O_3_^−^·2H_2_O
*M*_r_	292.68
Crystal system, space group	Monoclinic, *P*2_1_/*c*
Temperature (K)	150
*a*, *b*, *c* (Å)	18.8228 (10), 6.9495 (4), 19.3563 (10)
β (°)	99.817 (5)
*V* (Å^3^)	2494.9 (2)
*Z*	8
Radiation type	Mo *K*α
μ (mm^−1^)	0.33
Crystal size (mm)	0.30 × 0.20 × 0.20

Data collection	
Diffractometer	SuperNova, Dual, Cu at home/near, Atlas
Absorption correction	Multi-scan (*CrysAlis PRO*; Rigaku OD, 2019 ▸)
*T*_min_, *T*_max_	0.812, 1.000
No. of measured, independent and observed [*I* > 2σ(*I*)] reflections	13946, 6542, 5147
*R*_int_	0.023
(sin θ/λ)_max_ (Å^−1^)	0.712

Refinement	
*R*[*F*^2^ > 2σ(*F*^2^)], *wR*(*F*^2^), *S*	0.040, 0.108, 1.04
No. of reflections	6542
No. of parameters	343
H-atom treatment	H-atom parameters constrained
Δρ_max_, Δρ_min_ (e Å^−3^)	0.33, −0.30

Computer programs: *CrysAlis PRO* (Rigaku OD, 2019 ▸), *SHELXT2018/2* (Sheldrick, 2015*a* ▸), *SHELXL2018/3* (Sheldrick, 2015*b* ▸), *PLATON* (Spek, 2020 ▸), *Mercury* (Macrae *et al.*, 2020 ▸), *POVRay* (Cason, 2004 ▸), *PLATON* (Spek, 2020 ▸) and *publCIF* (Westrip, 2010 ▸).

## full crystallographic data

### 2-Amino-5-chloropyridin-1-ium 2,4,6-trioxo-1,3-diazinan-5-ide dihydrate Crystal data

**Table:**

C_5_H_6_ClN_2_^+^·C_4_H_3_N_2_O_3_^−^·2H_2_O	*F*(000) = 1216
*M**_r_* = 292.68	*D*_x_ = 1.558 Mg m^−^^3^
Monoclinic, *P*2_1_/*c*	Mo *K*α radiation, λ = 0.71073 Å
Hall symbol: -P 2ybc	Cell parameters from 4971 reflections
*a* = 18.8228 (10) Å	θ = 3.6–30.0°
*b* = 6.9495 (4) Å	µ = 0.33 mm^−^^1^
*c* = 19.3563 (10) Å	*T* = 150 K
β = 99.817 (5)°	Prism, yellow
*V* = 2494.9 (2) Å^3^	0.30 × 0.20 × 0.20 mm
*Z* = 8	

### 2-Amino-5-chloropyridin-1-ium 2,4,6-trioxo-1,3-diazinan-5-ide dihydrate Data collection

**Table:**

SuperNova, Dual, Cu at home/near, Atlas diffractometer	6542 independent reflections
Radiation source: micro-focus sealed X-ray tube	5147 reflections with *I* > 2σ(*I*)
SuperNova (Mo) X-ray Source monochromator	*R*_int_ = 0.023
Detector resolution: 10.4933 pixels mm^-1^	θ_max_ = 30.4°, θ_min_ = 2.6°
ωw scans	*h* = −24→24
Absorption correction: multi-scan (CrysAlis PRO; Rigaku OD, 2019)	*k* = −9→6
*T*_min_ = 0.812, *T*_max_ = 1.000	*l* = −18→25
13946 measured reflections	

### 2-Amino-5-chloropyridin-1-ium 2,4,6-trioxo-1,3-diazinan-5-ide dihydrate Refinement

**Table:**

Refinement on *F*^2^	0 restraints
Least-squares matrix: full	Hydrogen site location: mixed
*R*[*F*^2^ > 2σ(*F*^2^)] = 0.040	H-atom parameters constrained
*wR*(*F*^2^) = 0.108	W = 1/[Σ^2^(*F*O^2^) + (0.0476*P*)^2^ + 0.6984*P*] WHERE *P* = (*F*O^2^ + 2*F*C^2^)/3
*S* = 1.04	(Δ/σ)_max_ = 0.001
6542 reflections	Δρ_max_ = 0.33 e Å^−^^3^
343 parameters	Δρ_min_ = −0.30 e Å^−^^3^

### 2-Amino-5-chloropyridin-1-ium 2,4,6-trioxo-1,3-diazinan-5-ide dihydrate Special details

**Table:**

Geometry. Bond distances, angles etc. have been calculated using the rounded fractional coordinates. All su's are estimated from the variances of the (full) variance-covariance matrix. The cell esds are taken into account in the estimation of distances, angles and torsion angles
Refinement. Refinement on F^2^ for ALL reflections except those flagged by the user for potential systematic errors. Weighted R-factors wR and all goodnesses of fit S are based on F^2^, conventional R-factors R are based on F, with F set to zero for negative F^2^. The observed criterion of F^2^ > 2sigma(F^2^) is used only for calculating -R-factor-obs etc. and is not relevant to the choice of reflections for refinement. R-factors based on F^2^ are statistically about twice as large as those based on F, and R-factors based on ALL data will be even larger.The H atoms of the –NH_2_ groups were located from a difference Fourier map and refined freely. The other H atoms were placed geometrically and refined using a riding model.

### 2-Amino-5-chloropyridin-1-ium 2,4,6-trioxo-1,3-diazinan-5-ide dihydrate Fractional atomic coordinates and isotropic or equivalent isotropic displacement parameters (Å^2^)

**Table:**

	*x*	*y*	*z*	*U*_iso_*/*U*_eq_	
Cl1	0.87257 (2)	0.58875 (6)	0.79957 (2)	0.0262 (1)	
Cl2	0.01456 (2)	0.65022 (7)	0.64189 (2)	0.0331 (1)	
O1	0.48161 (6)	0.29532 (19)	0.55832 (5)	0.0241 (3)	
O2	0.24377 (6)	0.15226 (18)	0.50080 (5)	0.0232 (3)	
O3	0.33892 (6)	0.45699 (17)	0.71542 (5)	0.0209 (3)	
N5	0.40902 (7)	0.3677 (2)	0.63664 (6)	0.0172 (3)	
N6	0.36206 (6)	0.2313 (2)	0.52973 (6)	0.0180 (3)	
O4	0.39978 (6)	0.39067 (19)	0.89731 (6)	0.0272 (4)	
O5	0.63610 (6)	0.23191 (18)	0.95562 (5)	0.0229 (3)	
O6	0.53956 (6)	0.46773 (18)	0.73026 (5)	0.0216 (3)	
C11	0.42087 (8)	0.2982 (2)	0.57385 (7)	0.0178 (4)	
C12	0.29255 (8)	0.2292 (2)	0.54502 (7)	0.0178 (4)	
C13	0.28334 (8)	0.3118 (2)	0.60855 (7)	0.0190 (4)	
C14	0.34145 (8)	0.3827 (2)	0.65576 (7)	0.0173 (4)	
N7	0.47177 (7)	0.4344 (2)	0.81589 (6)	0.0189 (4)	
N8	0.51837 (7)	0.3158 (2)	0.92568 (6)	0.0199 (4)	
C15	0.46008 (8)	0.3809 (2)	0.88069 (8)	0.0192 (4)	
C16	0.58734 (8)	0.2981 (2)	0.90937 (8)	0.0178 (4)	
C17	0.59555 (8)	0.3545 (2)	0.84172 (7)	0.0190 (4)	
C18	0.53785 (8)	0.4195 (2)	0.79346 (7)	0.0177 (4)	
N1	0.67547 (7)	0.4856 (2)	0.70029 (6)	0.0192 (4)	
N2	0.62458 (7)	0.3966 (2)	0.58823 (7)	0.0261 (4)	
C1	0.68229 (8)	0.4424 (2)	0.63372 (8)	0.0196 (4)	
C2	0.75242 (8)	0.4493 (2)	0.61612 (8)	0.0217 (5)	
C3	0.81029 (8)	0.4945 (2)	0.66553 (8)	0.0220 (4)	
C4	0.79994 (8)	0.5347 (2)	0.73472 (8)	0.0199 (4)	
C5	0.73289 (8)	0.5300 (2)	0.75058 (8)	0.0199 (4)	
N3	0.20843 (7)	0.53881 (19)	0.74635 (6)	0.0188 (4)	
N4	0.25572 (7)	0.4593 (2)	0.86099 (7)	0.0244 (4)	
C6	0.19906 (8)	0.5027 (2)	0.81288 (8)	0.0193 (4)	
O7	0.77655 (6)	0.2806 (2)	0.93547 (6)	0.0325 (4)	
C7	0.12861 (9)	0.5152 (3)	0.82787 (8)	0.0253 (5)	
C8	0.07259 (9)	0.5605 (3)	0.77660 (8)	0.0265 (5)	
C9	0.08531 (9)	0.5963 (2)	0.70812 (8)	0.0224 (5)	
C10	0.15318 (9)	0.5862 (2)	0.69438 (8)	0.0217 (4)	
O8	0.09636 (6)	0.2133 (2)	0.49087 (6)	0.0346 (4)	
O9	−0.00582 (7)	0.1255 (2)	0.57464 (7)	0.0385 (4)	
O10	0.11145 (7)	0.6003 (2)	0.47080 (7)	0.0395 (4)	
H5A	0.44601	0.40394	0.66680	0.0210*	
H6	0.36970	0.18200	0.48947	0.0220*	
H13	0.23625	0.31983	0.61984	0.0230*	
H7A	0.43332	0.46700	0.78672	0.0230*	
H8A	0.51061	0.27394	0.96639	0.0240*	
H17	0.64191	0.34797	0.82868	0.0230*	
H1	0.63257	0.47895	0.71181	0.0230*	
H2	0.75892	0.42205	0.56951	0.0260*	
H2A	0.58118	0.38803	0.60077	0.0310*	
H2B	0.62828	0.35503	0.54527	0.0310*	
H3	0.85722	0.49913	0.65372	0.0260*	
H5	0.72573	0.55782	0.79696	0.0240*	
H3A	0.25032	0.51691	0.73631	0.0230*	
H4A	0.29875	0.44901	0.85025	0.0290*	
H4B	0.24985	0.43301	0.90465	0.0290*	
H7	0.12025	0.49171	0.87417	0.0300*	
H8	0.02506	0.56796	0.78680	0.0320*	
H10	0.16226	0.61216	0.64850	0.0260*	
H7B	0.73332	0.24406	0.93811	0.0490*	
H7C	0.77982	0.39306	0.94911	0.0490*	
H8B	0.09589	0.33708	0.48869	0.0520*	
H8C	0.14319	0.18508	0.50149	0.0520*	
H9A	0.03030	0.14603	0.55342	0.0580*	
H9B	−0.02290	0.01903	0.55642	0.0580*	
H10A	0.07720	0.68196	0.45889	0.0590*	
H10B	0.14490	0.65296	0.50189	0.0590*	

### 2-Amino-5-chloropyridin-1-ium 2,4,6-trioxo-1,3-diazinan-5-ide dihydrate Atomic displacement parameters (Å^2^)

**Table:**

	*U*^11^	*U*^22^	*U*^33^	*U*^12^	*U*^13^	*U*^23^
Cl1	0.0180 (2)	0.0300 (2)	0.0287 (2)	−0.0023 (2)	−0.0017 (1)	−0.0038 (2)
Cl2	0.0236 (2)	0.0411 (3)	0.0310 (2)	0.0059 (2)	−0.0059 (2)	0.0034 (2)
O1	0.0148 (5)	0.0390 (7)	0.0196 (5)	−0.0032 (5)	0.0062 (4)	−0.0073 (5)
O2	0.0161 (5)	0.0331 (7)	0.0197 (5)	−0.0034 (5)	0.0015 (4)	−0.0030 (5)
O3	0.0169 (5)	0.0315 (7)	0.0152 (5)	0.0011 (5)	0.0052 (4)	−0.0026 (5)
N5	0.0130 (6)	0.0243 (7)	0.0144 (5)	−0.0011 (5)	0.0026 (4)	−0.0022 (5)
N6	0.0146 (6)	0.0263 (7)	0.0136 (5)	−0.0008 (5)	0.0040 (4)	−0.0027 (5)
O4	0.0157 (5)	0.0467 (8)	0.0205 (5)	0.0053 (5)	0.0072 (4)	0.0080 (5)
O5	0.0160 (5)	0.0336 (7)	0.0186 (5)	0.0033 (5)	0.0018 (4)	0.0047 (5)
O6	0.0168 (5)	0.0346 (7)	0.0138 (5)	0.0001 (5)	0.0042 (4)	0.0025 (5)
C11	0.0160 (7)	0.0216 (8)	0.0163 (7)	0.0001 (6)	0.0038 (5)	−0.0005 (6)
C12	0.0151 (7)	0.0221 (8)	0.0166 (7)	0.0003 (6)	0.0035 (5)	0.0039 (6)
C13	0.0139 (7)	0.0253 (9)	0.0184 (7)	0.0010 (6)	0.0048 (5)	0.0017 (6)
C14	0.0173 (7)	0.0211 (8)	0.0145 (6)	0.0016 (6)	0.0057 (5)	0.0030 (6)
N7	0.0149 (6)	0.0278 (8)	0.0141 (6)	0.0025 (5)	0.0028 (4)	0.0031 (5)
N8	0.0165 (6)	0.0299 (8)	0.0139 (6)	0.0022 (6)	0.0047 (5)	0.0056 (6)
C15	0.0156 (7)	0.0250 (8)	0.0177 (7)	0.0004 (6)	0.0050 (5)	0.0013 (6)
C16	0.0149 (7)	0.0200 (8)	0.0190 (7)	0.0005 (6)	0.0044 (5)	0.0002 (6)
C17	0.0140 (7)	0.0250 (8)	0.0187 (7)	0.0000 (6)	0.0052 (5)	0.0007 (6)
C18	0.0162 (7)	0.0207 (8)	0.0170 (7)	−0.0028 (6)	0.0055 (5)	−0.0030 (6)
N1	0.0150 (6)	0.0256 (7)	0.0180 (6)	−0.0013 (5)	0.0059 (5)	−0.0010 (6)
N2	0.0182 (7)	0.0417 (9)	0.0190 (6)	−0.0050 (6)	0.0047 (5)	−0.0073 (6)
C1	0.0186 (7)	0.0216 (8)	0.0190 (7)	−0.0008 (6)	0.0045 (6)	0.0000 (6)
C2	0.0211 (8)	0.0256 (9)	0.0202 (7)	−0.0011 (6)	0.0091 (6)	−0.0025 (7)
C3	0.0165 (7)	0.0230 (8)	0.0283 (8)	−0.0016 (6)	0.0090 (6)	−0.0021 (7)
C4	0.0172 (7)	0.0187 (8)	0.0228 (7)	−0.0017 (6)	0.0010 (6)	−0.0005 (6)
C5	0.0204 (8)	0.0221 (8)	0.0172 (7)	−0.0002 (6)	0.0030 (5)	−0.0018 (6)
N3	0.0155 (6)	0.0226 (7)	0.0193 (6)	0.0012 (5)	0.0061 (5)	0.0011 (5)
N4	0.0171 (7)	0.0359 (9)	0.0206 (6)	0.0036 (6)	0.0040 (5)	0.0044 (6)
C6	0.0181 (7)	0.0214 (8)	0.0189 (7)	0.0005 (6)	0.0046 (5)	−0.0012 (6)
O7	0.0206 (6)	0.0332 (7)	0.0451 (7)	−0.0004 (5)	0.0095 (5)	−0.0077 (6)
C7	0.0195 (8)	0.0368 (10)	0.0211 (7)	0.0022 (7)	0.0080 (6)	0.0024 (7)
C8	0.0168 (8)	0.0342 (10)	0.0295 (8)	0.0017 (7)	0.0070 (6)	0.0007 (8)
C9	0.0189 (8)	0.0225 (8)	0.0245 (8)	0.0024 (6)	0.0000 (6)	0.0009 (7)
C10	0.0240 (8)	0.0225 (8)	0.0187 (7)	0.0006 (6)	0.0040 (6)	0.0021 (6)
O8	0.0204 (6)	0.0406 (8)	0.0424 (7)	0.0008 (6)	0.0046 (5)	0.0027 (6)
O9	0.0387 (8)	0.0413 (8)	0.0380 (7)	0.0019 (6)	0.0135 (6)	0.0028 (6)
O10	0.0297 (7)	0.0445 (9)	0.0434 (7)	0.0032 (6)	0.0039 (6)	−0.0042 (7)

### 2-Amino-5-chloropyridin-1-ium 2,4,6-trioxo-1,3-diazinan-5-ide dihydrate Geometric parameters (Å, º)

**Table:**

Cl1—C4	1.7322 (16)	C1—C2	1.419 (2)
Cl2—C9	1.7249 (17)	N2—H2A	0.8900
O1—C11	1.2309 (19)	C2—C3	1.358 (2)
O2—C12	1.2624 (18)	N2—H2B	0.8900
O3—C14	1.2732 (17)	C3—C4	1.414 (2)
N5—C11	1.3611 (18)	C4—C5	1.349 (2)
N5—C14	1.388 (2)	C2—H2	0.9500
N6—C11	1.3596 (19)	C3—H3	0.9500
N6—C12	1.3899 (19)	N3—C10	1.358 (2)
O4—C15	1.2332 (19)	N3—C6	1.3527 (19)
N5—H5A	0.8700	N4—C6	1.326 (2)
O5—C16	1.2551 (19)	C5—H5	0.9500
O6—C18	1.2741 (17)	N3—H3A	0.8600
N6—H6	0.8900	N4—H4B	0.8900
C12—C13	1.3944 (19)	N4—H4A	0.8700
C13—C14	1.391 (2)	C6—C7	1.408 (2)
N7—C15	1.3616 (19)	C7—C8	1.356 (2)
N7—C18	1.389 (2)	C8—C9	1.409 (2)
N8—C15	1.357 (2)	C9—C10	1.350 (2)
N8—C16	1.393 (2)	C7—H7	0.9500
C13—H13	0.9500	O7—H7C	0.8200
N7—H7A	0.8700	O7—H7B	0.8600
N8—H8A	0.8800	C8—H8	0.9500
C16—C17	1.400 (2)	C10—H10	0.9500
C17—C18	1.382 (2)	O8—H8B	0.8600
N1—C1	1.3503 (19)	O8—H8C	0.8900
N1—C5	1.361 (2)	O9—H9A	0.8600
N2—C1	1.316 (2)	O9—H9B	0.8600
C17—H17	0.9500	O10—H10A	0.8600
N1—H1	0.8700	O10—H10B	0.8700
			
C11—N5—C14	124.19 (13)	C1—N1—H1	118.00
C11—N6—C12	124.41 (12)	C1—N2—H2A	122.00
C11—N5—H5A	118.00	C1—N2—H2B	121.00
C14—N5—H5A	118.00	C1—C2—C3	120.39 (14)
C11—N6—H6	117.00	H2A—N2—H2B	117.00
C12—N6—H6	119.00	C2—C3—C4	119.29 (14)
O1—C11—N6	122.12 (13)	C3—C4—C5	119.74 (14)
N5—C11—N6	116.26 (13)	Cl1—C4—C3	120.67 (12)
O1—C11—N5	121.62 (13)	Cl1—C4—C5	119.58 (12)
O2—C12—N6	117.54 (12)	N1—C5—C4	120.16 (14)
O2—C12—C13	125.94 (14)	C1—C2—H2	120.00
N6—C12—C13	116.52 (13)	C3—C2—H2	120.00
C12—C13—C14	121.55 (14)	C2—C3—H3	120.00
O3—C14—C13	126.36 (14)	C4—C3—H3	120.00
N5—C14—C13	116.88 (12)	C6—N3—C10	122.84 (14)
O3—C14—N5	116.75 (13)	N1—C5—H5	120.00
C15—N7—C18	124.15 (13)	C4—C5—H5	120.00
C15—N8—C16	124.54 (12)	C10—N3—H3A	120.00
C14—C13—H13	119.00	C6—N3—H3A	117.00
C12—C13—H13	119.00	C6—N4—H4A	121.00
C15—N7—H7A	115.00	H4A—N4—H4B	119.00
C18—N7—H7A	120.00	C6—N4—H4B	120.00
C15—N8—H8A	117.00	N3—C6—C7	117.72 (14)
C16—N8—H8A	119.00	N3—C6—N4	119.42 (14)
O4—C15—N8	122.03 (14)	N4—C6—C7	122.86 (14)
N7—C15—N8	116.25 (13)	C6—C7—C8	120.38 (15)
O4—C15—N7	121.72 (14)	C7—C8—C9	119.60 (16)
O5—C16—N8	117.85 (13)	C8—C9—C10	119.63 (15)
O5—C16—C17	125.85 (14)	Cl2—C9—C8	120.28 (13)
N8—C16—C17	116.29 (13)	Cl2—C9—C10	120.09 (12)
C16—C17—C18	121.61 (14)	N3—C10—C9	119.82 (14)
O6—C18—C17	126.11 (14)	C6—C7—H7	120.00
N7—C18—C17	117.09 (12)	C8—C7—H7	120.00
O6—C18—N7	116.80 (13)	H7B—O7—H7C	106.00
C1—N1—C5	122.67 (13)	C7—C8—H8	120.00
C16—C17—H17	119.00	C9—C8—H8	120.00
C18—C17—H17	119.00	N3—C10—H10	120.00
N1—C1—N2	119.38 (14)	C9—C10—H10	120.00
N1—C1—C2	117.72 (14)	H8B—O8—H8C	103.00
N2—C1—C2	122.90 (14)	H9A—O9—H9B	103.00
C5—N1—H1	119.00	H10A—O10—H10B	109.00
			
C14—N5—C11—O1	176.94 (14)	C16—C17—C18—O6	−177.54 (14)
C14—N5—C11—N6	−3.5 (2)	C16—C17—C18—N7	2.7 (2)
C11—N5—C14—O3	−177.60 (13)	C5—N1—C1—N2	178.47 (14)
C11—N5—C14—C13	3.6 (2)	C5—N1—C1—C2	−1.7 (2)
C12—N6—C11—O1	179.22 (14)	C1—N1—C5—C4	0.9 (2)
C12—N6—C11—N5	−0.4 (2)	N1—C1—C2—C3	1.2 (2)
C11—N6—C12—O2	−175.86 (14)	N2—C1—C2—C3	−178.93 (14)
C11—N6—C12—C13	3.7 (2)	C1—C2—C3—C4	0.0 (2)
O2—C12—C13—C14	176.03 (14)	C2—C3—C4—Cl1	178.85 (11)
N6—C12—C13—C14	−3.5 (2)	C2—C3—C4—C5	−0.8 (2)
C12—C13—C14—O3	−178.55 (14)	Cl1—C4—C5—N1	−179.26 (11)
C12—C13—C14—N5	0.1 (2)	C3—C4—C5—N1	0.4 (2)
C18—N7—C15—O4	−177.42 (14)	C10—N3—C6—N4	−179.28 (14)
C18—N7—C15—N8	2.4 (2)	C10—N3—C6—C7	0.1 (2)
C15—N7—C18—O6	177.04 (14)	C6—N3—C10—C9	−0.9 (2)
C15—N7—C18—C17	−3.2 (2)	N3—C6—C7—C8	0.5 (3)
C16—N8—C15—O4	178.69 (14)	N4—C6—C7—C8	179.89 (17)
C16—N8—C15—N7	−1.1 (2)	C6—C7—C8—C9	−0.4 (3)
C15—N8—C16—O5	−178.53 (14)	C7—C8—C9—Cl2	178.93 (15)
C15—N8—C16—C17	0.7 (2)	C7—C8—C9—C10	−0.4 (3)
O5—C16—C17—C18	177.64 (14)	Cl2—C9—C10—N3	−178.31 (11)
N8—C16—C17—C18	−1.6 (2)	C8—C9—C10—N3	1.0 (2)

### 2-Amino-5-chloropyridin-1-ium 2,4,6-trioxo-1,3-diazinan-5-ide dihydrate Hydrogen-bond geometry (Å, º)

**Table:**

*D*—H···*A*	*D*—H	H···*A*	*D*···*A*	*D*—H···*A*
N1—H1···O6	0.87	1.85	2.7207 (18)	176
N2—H2*A*···O1	0.89	2.02	2.7461 (18)	138
N2—H2*B*···O5^i^	0.89	1.87	2.7610 (17)	180
N3—H3*A*···O3	0.86	1.83	2.6863 (18)	177
N4—H4*A*···O4	0.87	2.00	2.7248 (18)	139
N4—H4*B*···O2^ii^	0.89	1.97	2.8606 (17)	173
N5—H5*A*···O6	0.87	2.02	2.8786 (17)	173
N6—H6···O4^i^	0.89	2.03	2.9005 (17)	170
N7—H7*A*···O3	0.87	2.06	2.8985 (17)	163
N8—H8*A*···O1^ii^	0.88	2.01	2.8762 (16)	172
O7—H7*B*···O5	0.86	1.92	2.7581 (16)	164
O7—H7*C*···O2^iii^	0.82	2.13	2.9155 (18)	160
O8—H8*B*···O10	0.86	1.89	2.739 (2)	167
O8—H8*C*···O2	0.89	1.91	2.7810 (16)	165
O9—H9*A*···O8	0.86	1.93	2.7864 (18)	168
O10—H10*B*···O7^iii^	0.87	1.96	2.8287 (18)	175
C10—H10···O7^iii^	0.95	2.44	3.321 (2)	153

Symmetry codes: (i) *x*, −*y*+1/2, *z*−1/2; (ii) *x*, −*y*+1/2, *z*+1/2; (iii) −*x*+1, *y*+1/2, −*z*+3/2.
